# An isolated anterior mitral leaflet cleft: a case report

**DOI:** 10.1186/1476-7120-8-26

**Published:** 2010-07-13

**Authors:** Giovanni Minardi, Stefania Leonetti, Leda Bernardi, Giovanni Pulignano, Paolo Giuseppe Pino, Lidia Boccardi, Carla Manzara, Francesco Musumeci

**Affiliations:** 1 Cardiodiagnostica non invasiva, Department of Cardiology and Cardiovascular Surgery, Azienda Ospedaliera San Camillo-Forlanini, Rome, Italy

## Abstract

**Introduction:**

The anterior mitral leaflet cleft is an unusual congenital lesion most often encountered in association with other congenital heart defects. The isolated anterior leaflet cleft is quite a rare anomaly and is usually cause of mitral valve regurgitation. The importance of the lesion is that it is often correctable. When feasible, cleft suture and, eventually, annuloplasty are preferable to valve replacement. Echocardiography is the first choice technique in the evaluation of mitral valve disease, providing useful information about valve anatomy and hemodynamic parameters.

**Case presentation:**

We present a case of an isolated anterior mitral leaflet cleft producing moderate-severe mitral regurgitation correctly identified by echocardiography and successfully surgically corrected.

**Conclusion:**

Isolated cleft is a rare aberration, that has to be known in order to be diagnosed. Transthoracic and transesophageal echocardiography is the most useful non invasive technique for cleft diagnosis and to indicate the right surgical correction.

## Introduction

The anterior mitral leaflet cleft is an unusual congenital lesion first described in 1954 [1].

Most often, this lesion is encountered in association with other congenital heart defects (ventricular septal defect, tetralogy of Fallot, transposition of great arteries, double-outlet right ventricle, tricuspid atresia and double-inlet left ventricle) [2].

The isolated anterior leaflet cleft is quite a rare anomaly, in which some anatomic data are specific: unlike endocardial cushion defect, mitral annulus is in normal position, cleft pointed towards left ventricular outflow tract, mitral and tricuspid valves are, as in normal subject, attached to the interventricular septum at different levels (tricuspid valve junction is lower than mitral valve junction) [1].

Mitral cleft is usually cause of isolated valve regurgitation (MR). This lesion is important because it is often correctable.

When feasible, cleft suture and, eventually, annuloplasty are preferable to valve replacement.

Sometimes, direct cleft suture is not possible because of the lack of valvular tissue; in these cases glutaraldehyde-treated autologus pericardium may be used [1].

We here present a clinical case of isolated anterior mitral leaflet cleft producing moderate-severe mitral regurgitation correctly identified by echocardiography.

## Case presentation

A 27 year old Italian woman was referred to our hospital with suspected congenital heart disease. At admission the patient was asymptomatic for dyspnea and chest pain. Auscultation showed a 2-3/6 grade holosystolic murmur on the left sternal border. ECG revealed no significant abnormalities. A previous transthoracic echocardiography (TTE) had revealed a dysplastic mitral valve with moderate regurgitation (eccentric jet towards left atrium lateral wall), a mild left ventricle enlargement (end-diastolic diameter 57 mm, end-systolic diameter 38 mm) with a conserved systolic function (LVEF 70%), an intact interatrial septum, and normal systolic pulmonary artery pressure.

A transesophageal echocardiography (TEE) had confirmed the mitral valve dysplasia (thick and redundant leaflets), with A2-P2 prolapse and moderate valve regurgitation.

**Figure 1:**
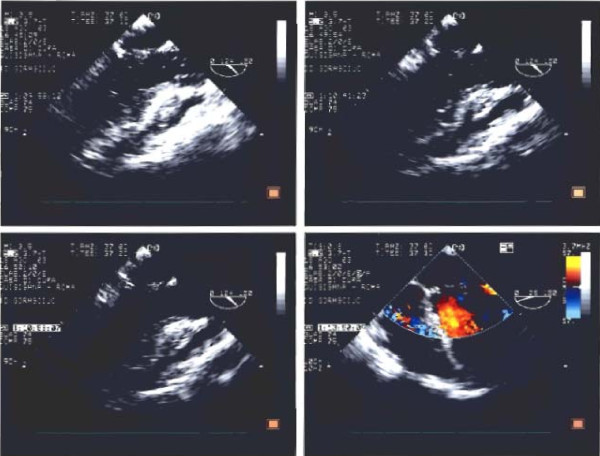
**Transesophageal echocardiographic view of mitral valve: valve dysplasia and anterior leaflet cleft are showed**.

**Figure 2:**
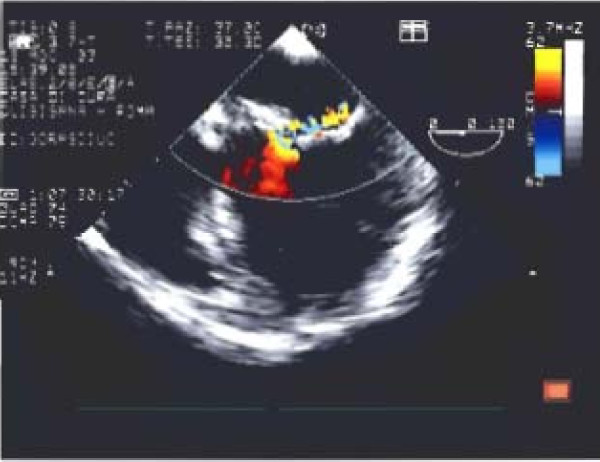
**Transesophageal echocardiographic view of mitral valve: moderate-severe mitral regurgitation with jet originating centrally and directed towards lateral wall of the left atrium is seen**.

TTE and TEE, performed in our echolab confirmed mitral dysplasia and P2 prolapse but showed mitral anterior leaflet cleft, mitral annulus dilatation and moderate-severe MR with a holosystolic jet originating centrally and then directed towards lateral wall of the left atrium (jet area 10 cm2, jet/left atrium ratio 0,52) (Figs 1,2). Systolic inversion of pulmonary venous flow, left atrium enlargement and moderate pulmonary systolic hypertension were also found. The patient was addressed to surgery and underwent mitral valve repair, with directed cleft suture and posterior ring annuloplasty. Intraoperative TEE showed no residual MR, nor left ventricular outflow obstruction.

Postoperative TTE confirmed good results of mitral valve repair, a normal LVEF and the absence of pericardial effusion.

The postoperative course was uneventful and the patient was discharged.

## Discussion

Clefts, defined as slit-like holes or defects, are hypothesized as being a result of incomplete expression of an endocardial cushion defect which most commonly involves the anterior mitral valve leaflet with a paediatric incidence of 1:1340 [3]. This lesion is uncommon in adults and is responsible for 33% of congenital mitral valve regurgitation [4].

Nevertheless, if atrio-ventricular junction is normal and MR is mild, patients may be asymptomatic for many years and mitral cleft may be found by chance.

Cleft is the main determinant of MR, but often annular dilatation and restricted motion of anterior leaflet coexist, contributing to MR.

Regurgitation degree is consequence of interactions between leaflets, accessory chordal attachment, papillary muscles, left atrium and left ventricle free wall [5].

Echocardiography is the first choice technique in the evaluation of suspected or known mitral valve congenital abnormalities, providing useful information about valve anatomical and morphological details, mechanism of MR and its quantitative evaluation.

Sometimes, preoperative cleft diagnosis is difficult because of the position, dimensions and shape of the lesion [6]. In these patients three dimensional echocardiography (3DE) can be useful. 3DE enables precise assessment of mitral valve pathology as it provides a structural display in three dimension from every perspective. While its utility has been extensively documented in acquired mitral valve disease, data on its incremental value in congenital mitral valve pathology are sparse.

Real-time 3DE (RT3DE), both TTE and TEE imaging, can be highly sensitive in the diagnosis of cleft valves, providing accurate pathoanatomic definition, including the width and depth of the cleft, degree of fibrosis and edge retraction, presence of accessory chordae, origin, and mechanism of the regurgitant jet in addition to characterizing associated congenital malformations. Furthermore, RT3DE imaging also allows visualization of mitral valve en face either from the left atrium or left ventricle and provides a view of the valve similar to that seen intraoperatively by the cardiac surgeon [7].

TEE is, in any case, the gold standard technique to characterize mechanism and morphological details of mitral cleft. TEE has been shown to be extremely sensitive in the evaluation of mitral regurgitation. Assessment of MR by TEE appears to correlate closely with the angiographic degree of mitral regurgitation.

Satisfactory results have been obtained in 93% of surgical mitral repair performed on the basis of transesophageal evaluation [6].

TEE, showing the immediate results of repaired valve and the possible mechanism of suboptimal repair is very useful in intraoperative monitoring of repaired mitral valve, while TTE is mandatory during follow-up for the evaluation of the repaired valve, providing residual functional and anatomical data.

In our case echocardiography has been lessential for the diagnosis. The importance of identifyng and interpreting a mitralic disease justifies the need to perform some echocardiographic evaluations in echo-labs with suitable experience and competence.

At the moment no data are reported comparing echocardiography with any other imaging technique in the evaluation of isolated mitral cleft.

Cardiac magnetic resonance (CMR) has the potential role of correctly identifying this congenital lesion. No study is reported in literature. Neverthless CMR is not extensively available and its diagnostic utilization is not justifiable because of its cost.

The prognosis of repaired mitral cleft is usually excellent with reoperation rate of ~ 3,1% and a significant survival improvement [7].

When technically feasible, mitral repair is preferable to valve replacement [1].

Morbidity and late mortality have been shown to be not statistically different with an overall survival probability of 67 ± 7% at 5 years after repair versus 73 ± 9% after replacement [7].

## Conclusions

Isolated cleft is a rare aberration, that has to be known in order to be diagnosed. Echocardiography is the most useful not invasive technique for cleft diagnosis and to indicate the right surgical correction.

## Consent

Written informed consent was obtained from the patient for publication of this case report and accompanying images. A copy of the written consent is available for review by Editor-in-Chief of this journal.

## Competing interests

The authors declare that they have no competing interests.

## Authors' contributions

GM and PGP made substantial contributions to analyze and interpret the patient data; GM, LB, GP, and CM performed TTE and TEE; FM performed mitral valve repair; GM, SL and LB have been involved in drafting the manuscript and revising it critically. All authors have given final approval of the version to be published.
